# Impact of epicardial fat on the duration of radiofrequency energy delivery during catheter ablation of atrial fibrillation

**DOI:** 10.1016/j.ijcha.2020.100555

**Published:** 2020-06-10

**Authors:** Victor Oudin, Claude Marcus, Laurent Faroux, Madeline Espinosa, Damien Metz, François Lesaffre

**Affiliations:** a Department of Cardiology, Reims University Hospital, 51092 Reims cedex, France; b Department of Radiology, Reims University Hospital, 51092 Reims cedex, France

**Keywords:** Atrial fibrillation, Computed tomography, Epicardial fat, Radiofrequency ablation

## Abstract

**Aims:**

This study aimed to determine the impact of the volume of epicardial fat on the duration of radiofrequency (RF) energy delivery during the procedure of ablation of atrial fibrillation (AF).

**Methods:**

The volume of epicardial fat was measured from spiral computerized tomography scan. The primary endpoint was the duration of RF delivery for pulmonary vein isolation (PVI), and the overall total duration of RF application. Secondary endpoint was conversion of AF to sinus rhythm or organisation of the arrhythmia after PVI.

**Results:**

From March 2015 to May 2018, 222 patients (45.5% with persistent AF) underwent a first RF catheter ablation procedure for AF. The total duration of RF delivery, and the duration of RF delivery specifically for PVI were significantly associated with higher total volume of epicardial fat (p = 0.0002; p = 0.009 respectively), periatrial (p = 0.003; p = 0.045) and periventricular epicardial fat (p = 0.001; p = 0.012). In multivariate analysis, total epicardial fat volume was not significantly associated with total RF delivery duration (p = 0.743). For patients with arrhythmia at the time of the procedure, patients who achieved conversion or organisation of their arrhythmia after PVI had similar levels of total epicardial fat to those whose arrhythmia persisted (65 ± 35.2 vs 74.5 ± 31.2 ml; p = 0.192).

**Conclusion:**

We observed a significant relation between total, periatrial, and periventricular epicardial fat, and the duration of RF delivery during ablation of AF. This relation was not significant by multivariate analysis meaning that epicardial fat may be a marker, but not an independent factor, of ablation complexity.

## 1 Background

Atrial fibrillation (AF) is a heart rhythm disorder that affects around one quarter of adults in developed countries, and 600,000 patients in France. World prevalence of AF is estimated at between 0.4 and 1%, and is expected to double within 20 years. AF represents a major public health burden, and is associated with reduced quality of life, increased risk of stroke, and may also cause or aggravate left ventricular function, thus leading to heart failure, and increased mortality, especially from cardiovascular causes.

Catheter ablation of AF is a therapy indicated when anti-arrhythmic drugs fail in patients with paroxysmal or persistent AF who remain symptomatic according to the European Heart Rhythm Association symptom scale (Grade 1A recommendation) [1]. It can also be considered as first line therapy as an alternative to antiarrhythmic drugs, in selected patients with symptomatic paroxysmal AF (Grade IIa) [1]. Recent studies have widened the indication for catheter ablation of AF, notably to heart failure patients further to the CASTLE-AF study [2].

Epicardial fat (EF) is defined as adipose tissue deposited between the myocardium and the visceral layer of the pericardium. Its volume depends on the patient’s weight [3]. EF promotes fibrosis of the atrial myocardium via infiltration of free fatty acids. Furthermore, endocrine and paracrine effects [4], [5], which are as yet poorly elucidated, contribute to the significant relationship described between EF and incidence of AF [6]. The volume of EF has been shown to be significantly related to the recurrence of AF after radiofrequency (RF) catheter ablation, independently of the patient’s weight and of the volume of the left atrium (LA) [7], [8].

To the best of our knowledge, no study to date has investigated the link between the quantity of EF, and procedural parameters during RF ablation of AF (e.g. ablation time, conversion to sinus rhythm after pulmonary vein isolation, …). Such data would make it possible to broaden our understanding of the mechanisms by which EF contributes to the prevalence and recurrence of AF.

The main objective of this study was therefore to determine the impact of the volume of EF on the duration of RF energy delivery during the ablation procedure. Secondary objectives were to determine whether the volume of EF was associated with AF conversion to sinus rhythm, or organisation to another supraventricular arrhythmia (flutter, focal atrial tachycardia) after pulmonary vein isolation (PVI) in patients in arrhythmia at the time of the procedure; and finally, to investigate whether the volume of EF was associated with the recurrence of AF at one year.

## 2 Methods

### 2.1 Population

We performed an observational study and retrospectively included patients undergoing a first RF ablation procedure for valvular or nonvalvular AF between March 2015 and May 2018 in a single, large university hospital in Reims, north-eastern France. Non-inclusion criteria were as follows: a history of AF ablation; missing data from the ablation procedure; no available contrast computed tomography (CT) scan prior to ablation (e.g. in case of hyperthyroidism, allergy to iodine, severe renal insufficiency contraindicating the use of contrast medium or patient’s refusal to undergo the scan). The primary endpoint was the duration of RF energy delivery (total RF delivery time and RF delivery time specifically dedicated to PVI). Secondary endpoints were conversion of AF to sinus rhythm or to another type of atrial arrhythmia after PVI and recurrence of AF at one year follow-up.

### 2.2 Pre-procedural work-up

Thoracic CT scan was performed systematically in all patients prior to ablation. A 64-row General Electric Discovery CT750 HD system (GE Healthcare, Milwaukee, WI, USA) was used to evaluate the anatomy and volume of the LA as well as adjacent structures. The volume of EF was measured *a posteriori*. After one acquisition without contrast injection, the region of interest was located in the LA to enable image acquisition with contrast injection. Images were acquired at inhalation breath-hold, with the patient’s arms above the head, in the cranio-caudal direction, with the following parameters: ECG-gated, 1.25 mm slice thickness, 0.4 sec rotation time. Images were acquired from 2 cm above the pulmonary apex to 2 cm below the cardiac apex. Iodinated contrast medium was administered via an antebrachial vein (90 ml Iomeprol 350 mg iodine/ml, Bracco imaging, France) at 5 ml/sec followed by a bolus of 50 ml of saline at 4 ml/sec.

### 2.3 Radiofrequency ablation procedure

A minimum of 3 weeks of anticoagulant therapy was required prior to the procedure. During the procedure, a bolus of unfractionated heparin was administered after trans-septal puncture, and additional boluses were given as required to achieve an activated clotting time greater than 300 sec. All procedures were performed under general anaesthesia by the same operator in an interventional catheterization laboratory equipped with the 3D cartography system CARTO3® (Biosense Webster, Johnson & Johnson). The steerable decapolar Dynamic XT™ coronary sinus catheter (Boston Scientific) (6 French) was used as the atrial reference. The irrigated ablation NAVISTAR SMARTTOUCH® catheter (Johnson & Johnson Medical) (9 French) delivered the RF energy; a multipolar LASSO NAV or PENTARAY NAV® catheter (Biosense Webster, Johnson & Johnson Medical) (8 French) was used for electrophysiological mapping and to confirm PVI. Generator parameters were standardized: maximal temperature 48 °C, maximal RF power 30 Watts, 25 Watts on the posterior wall of the LA. Contact information was used (minimum 8 g, 40% stability time, 1.5 mm inter-lesion distance). The duration of RF energy for each point-by-point lesion was defined as the time required to obtain complete elimination of the positive component of the atrial electrogram, associated with a significant decrease in impedance, added to a standard consolidation application of 10 sec.

Ablation of paroxysmal AF consisted in wide antral isolation, 2 by 2, of the pulmonary veins, confirmed by the achievement of bidirectional conduction block. Inducibility of AF was tested at the end of the procedure by stimulation of the LA (minimum atrial cycle 200 ms or corresponding to the atrial refractory period). Only sustained common flutter, either induced or previously documented, was treated by RF.

For persistent AF, ablation consisted in wide antral ablation of the pulmonary veins, confirmed in sinus rhythm by achievement of bidirectional conduction block. Complementary treatment was only performed in case of persistent atrial arrhythmia after this step, and could include defragmentation, mapping and ablation of focal atrial tachycardia or flutter, as required. If the procedure failed to achieve conversion to sinus rhythm or organisation of the arrhythmia, then electric cardioversion was performed by antero-posterior external electric shock at 200 Joules. Anticoagulation was systematically prescribed for 3 months after the procedure, then adapted thereafter according to CHA2DS2-VASc score. Anti-arrhythmic therapy was mostly pursued for 3 months after the procedure, then adapted on a case-by-case basis thereafter.

### 2.4 CT data

The volume of periatrial and periventricular EF was measured retrospectively by an experienced radiologist from pre-procedure CT scans, using a postprocessing workstation (Advantage server, GE Healthcare, Milwaukee, WI, USA). The observer manually traced the pericardial contours. The upper boundary of analysis was the right pulmonary artery, and the lower boundary was the section below the posterior left anterior descending artery. Fat-containing voxels were identified by selecting attenuation values between −190 and −30 Hounsfield Units, as in previous studies [4]. After selecting the volume tool, the volume of EF contained in the selected area was calculated in mL, yielding the total volume of EF. The observer then isolated the atrial cavities by manual tracing to obtain the volume of periatrial and periventricular EF. The volume of the LA was also measured according to standardized procedures, excluding the left atrial appendage and the pulmonary veins.

### 2.5 Data recorded

Clinical, paraclinical and interventional data were recorded retrospectively. Body fat mass was calculated using the Deurenberg equation. All patients had a systematic follow-up visit at 3 months after the procedure, with a 24-hour Holter monitoring; then at 6–12 months after the procedure by their usual cardiologist. Clinical success of AF ablation at one year was defined as the absence of recurrent symptoms attributable to the arrhythmia during the 12 months following the procedure. Asymptomatic recurrence of AF was defined as a more-than-30-seconds episode of AF detected by ECG-Holter monitoring with a clinical success. We did not consider as recurrence any arrhythmias occurring within 3 months after the procedure (blanking period).

### 2.6 Statistical analysis

Quantitative variables are described as mean ± standard deviation, and categorical variables as number (percentage). Means were compared using the Student *t* test. Relations between quantitative variables were evaluated two-by-two using simple linear regression. Multivariable analysis was performed using a generalized linear model and included all variables of interest that were related to the total RF delivery time with a p-value <0.05. To take account of the risk of multiple collinearity between the variables body mass index (BMI), fat mass and lean mass, only body mass index was included in the multivariable model. Similarly, to take account of the risk of multiple collinearity between the variables paroxysmal AF and persistent AF, only paroxysmal AF was included in the multivariable model. All analyses were performed using SPSS version 21 (IBM SPSS Statistics, Chicago, USA).

## 3 Results

### 3.1 Study population

Between March 2015 and May 2018, a total of 252 RF ablation procedures for AF were performed in the University Hospital of Reims. Among these, 25 were excluded because the patients had previously undergone one or more AF ablation procedures. Five patients had no CT scan prior to the procedure due to contraindications. Thus, a total of 222 patients were included in the final analysis, of whom 101 had persistent AF.

The pre-procedural clinical, echocardiographic and biological characteristics of the patients are shown in Table 1. The total, periatrial and periventricular volume of epicardial fat was respectively 66.1 ± 29.5; 24.2 ± 12.8 and 41.8 ± 20.1 ml.

**Table 1:** Baseline characteristics of the study population.

Variable	N = 165
Meal sex	169 (76.1)
Age (years)	58.4 ± 9.5
Height (cm)	174 ± 8.46
Weight (kg)	87.9 ± 18.5
Body mass index (kg/m^2^)	28.9 ± 5.4
Fat mass index (%)	34 ± 7.9
Paroxysmal atrial fibrillation	121 (54.5)
Persistent atrial fibrillation	101 (45.5)
Long standing atrial fibrillation	15 (6.8)
Duration of atrial fibrillation (years)	4.1 ± 4.3
Hypertension	103 (46.4)
History of stroke	10 (4.5)
Diabetes	25 (11.3)
Glomerular filtration rate (ml/m^2^/1.7)	80.3 ± 16.4
Dyslipidemia	94 (42.3)
Conserved left ventricular ejection fraction	188 (84.7)
History of cavotricuspid isthmus ablation	35 (15.8)
Failed therapy with class Ic anti-arrhythmics	120 (54.1)
Failed therapy with class III anti-arrhythmics	156 (70.3)
Left atrial volume (ml)	120 ± 40.97
Total volume of epicardial fat (ml)	66.1 ± 29.46
Volume of periatrial epicardial fat (ml)	24.2 ± 12.75
Volume of periventricular epicardial fat (ml)	41.8 ± 20.06

Quantitative variables are described as mean ± standard deviation, and categorical variables as number (percentage)

The procedural characteristics are presented in Table 2. The duration of RF energy delivery for PVI and the total duration of RF energy delivery were respectively 1913 ± 482 and 2165 ± 609 sec. Overall, among the population of 222 patients, 88 (39.7%) had AF at the time of the procedure, of whom 23 were restored to sinus rhythm and in 6 patients, organisation of the arrhythmia was observed after PVI. At one year, patients with paroxysmal AF and with persistent AF had respectively 78.5% and 78.2% of clinical success. Asymptomatic recurrence of AF amount to 3.6%.

**Table 2:** Procedural data from the radiofrequency catheter ablation procedure.

Variable	N = 165
Treatment with Amiodarone	100 (45)
Treatment with Flecainide	72 (32.4)
Sinus rhythm before ablation	134 (60.3)
Complete pulmonary vein isolation	220 (99.1)
Reduction of atrial fibrillation after pulmonary vein isolation	23 (10.4)
Organisation of atrial fibrillation after pulmonary vein isolation	6 (2.7)
Cavotricuspid isthmus ablation	67 (30.2)
Mitral isthmus ablation	5 (2.3)
Defragmentation	43 (19.4)
Additional line ablation	37 (16.6)
Procedure duration (min)	136 ± 35.8
Total duration of radiofrequency energy delivery (seconds)	2165 ± 609
Duration of radiofrequency delivery for pulmonary vein isolation (seconds)	1913 ± 482
Power (Watt)	25.2 ± 2.6
Temperature (°C)	36.4 ± 2.9
External electric shock post-procedure	54 (24.3)
Sinus rhythm at the end of the procedure	220 (99.1)
Inducibility of atrial fibrillation (in 59.5% of patients)	11 (5)
Follow-up duration (months)	17 ± 6.8

Quantitative variables are described as mean ± standard deviation, and categorical variables as number (percentage).

### 3.2 Primary outcome

**Fig. 1:**
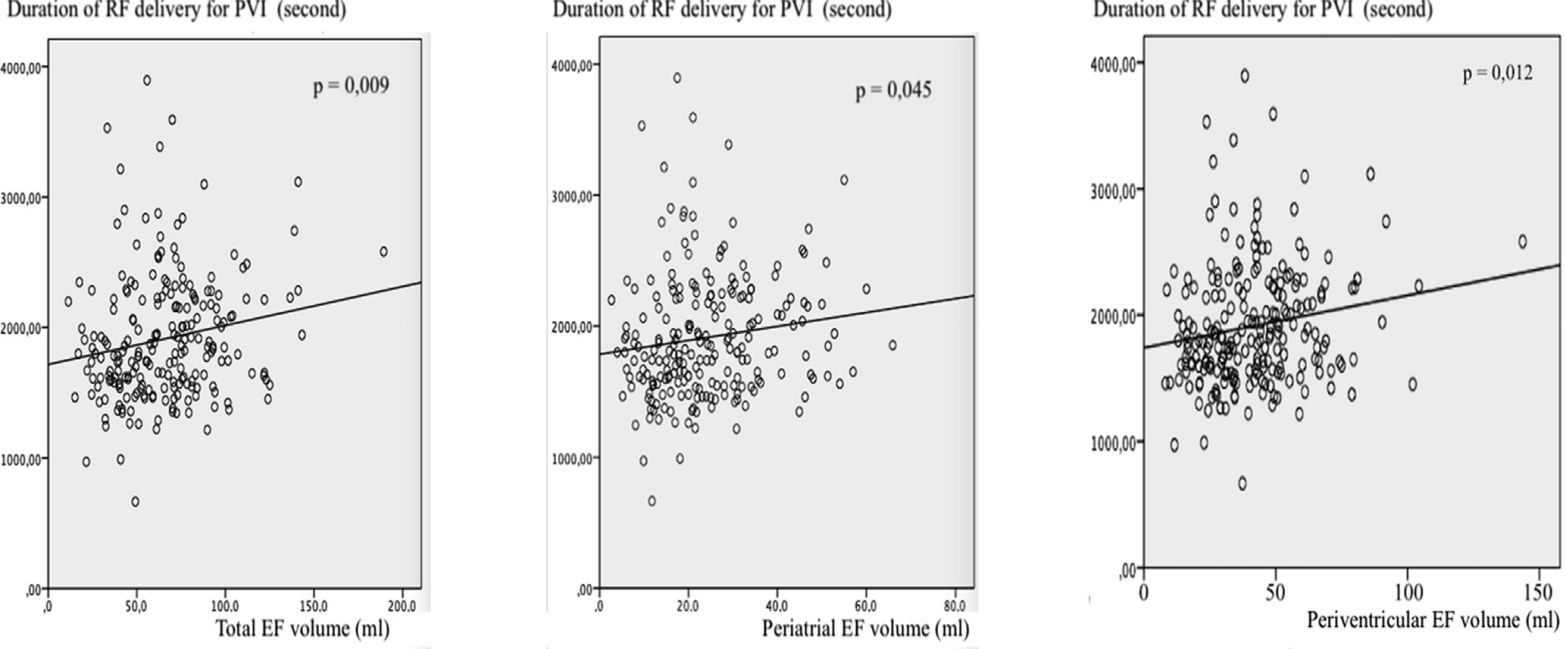
Relationship between epicardial fat, and duration of radiofrequency energy delivery required for isolation of the pulmonary veins.

**Fig. 2:**
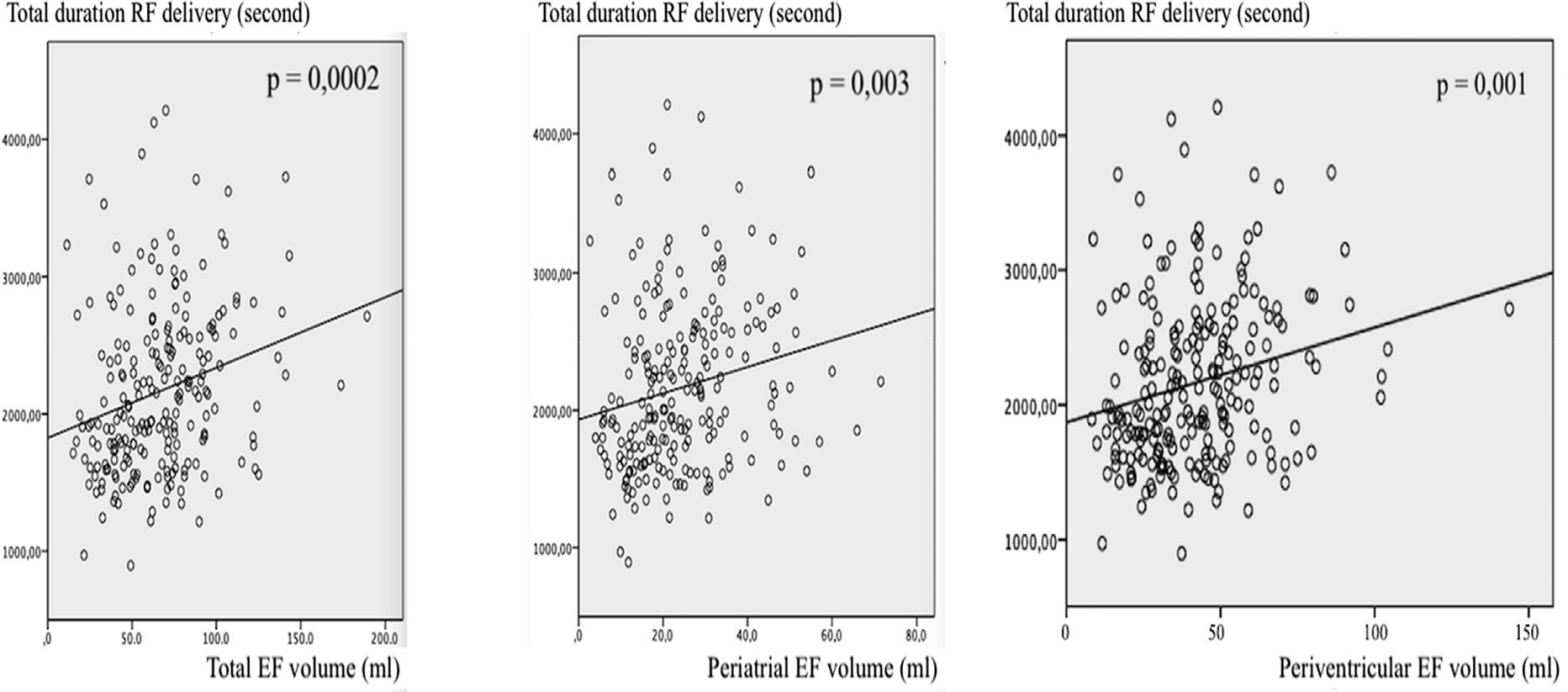
Relationship between epicardial fat, and the total duration of radiofrequency energy delivery.

Patients with persistent AF had significantly higher volumes of total EF (76.4 ± 32 vs 57.5 ± 24.1 ml; p < 0.0001), periatrial (28.6 ± 13.1 vs 20.6 ± 11.3 ml; p < 0.0001) and periventricular EF (47.7 ± 22.5 vs 36.9 ± 16.3 ml; p < 0.0001) as compared to patients with paroxysmal AF. The duration of RF delivery for PVI was significantly longer in patients with greater total (p = 0.009), periatrial (p = 0.045), and periventricular epicardial fat volume (p = 0.012) (Fig. 1). The total RF delivery duration was significantly associated with greater total (p = 0.0002), periatrial (p = 0.003), and periventricular epicardial fat volumes (p = 0.001) (Fig. 2).

Table 3 shows factors that were associated with the total duration of radiofrequency energy delivery in univariate and multivariate analysis; in multivariate analysis, the total volume of EF was no longer significantly associated with the total duration of RF delivery (Beta coefficient = 0.6; p = 0.743). Furthermore, in the paroxysmal and persistent AF subgroup, there is no significant association between the volume of EF and the total duration of RF delivery (Beta coefficient of the interaction term “Paroxysmal AF*Total epicardial fat volume” = −0.8; p = 0.760). We also observed a significant relationship between the total volume of EF and the LA volume (p < 0.0001; R^2^ = 0.176; VIF = 1.214).

**Table 3:** Factors associated with the total duration of radiofrequency energy delivery by univariate and multivariate analysis.

Factors associated with the total duration of radiofrequency energy delivery				
	Univariate analysis	Multivariate analysis		
Variable	p-Value	Beta coefficient	Standard error	p-Value
Male sex	0.004	169.4	98	0.084
Paroxysmal atrial fibrillation (AF)	<0.001	−296.4	203.8	0.146
Hypertension	0.179	–	–	–
History of stroke	0.588	–	–	–
Diabetes	0.801	–	–	–
Ischemic heart disease	0.205	–	–	–
History of cavotricuspid isthmus ablation	0.012	−201.9	109	0.064
Treatment with Amiodarone	0.021	–	–	–
Treatment with Flecainide	<0.001	–	–	–
Treatment with Calcium-channel blockers	0.586	–	–	–
Arrhythmia at the time of procedure	<0.001	–	–	–
External electric shock post-procedure	<0.001	–	–	–
History of cardiac surgery	0.538	–	–	–
Dyslipidemia	0.944	–	–	–
Treatment with Statins	0.627	–	–	–
Chronic obstructive pulmonary disease	0.371	–	–	–
Body mass index	0.001	14.7	8	0.068
Left atrial volume	0.005	1.8	1.1	0.102
Total epicardial fat volume	0.007	0.6	1.8	0.743
Paroxysmal AF*Total epicardial fat volume	–	−0.8	2.8	0.760

#### 3.3 Secondary outcomes

Among patients with arrhythmia at the time of the procedure, there was no significant relationship between epicardial fat volume, and conversion to sinus rhythm after PVI (64.1 ± 36 vs 73.9 ± 31.2 ml; p = 0.196). There was also no significant relationship when considering conversion to sinus rhythm or organisation of the arrhythmia after PVI (65 ± 35.2 vs 74.5 ± 31.2 ml; p = 0.192). The duration of RF delivery for PVI was significantly longer in patients with persistent AF as compared to patients with paroxysmal AF (2052 ± 523 vs 1797 ± 412 sec; p = 0.0001). About clinical results at one year after the ablation procedure, EF volume was not associated with symptomatic AF recurrence (64.9 ± 30.3 ml vs 66.9 ± 29.5 ml; p = 0.691) but was associated with asymptomatic AF recurrence (92.2 ± 46 ml vs 65.6 ± 28.5 ml; p = 0.012).

## 4 Discussion

Our study found a significant relationship between the volume of total, periatrial, and periventricular epicardial fat, and the duration of RF energy delivery during catheter ablation of AF. This relationship was no longer significant in multivariable analysis, indicating that EF may be a marker, but not an independent factor, of ablation complexity.

The lesion induced by RF ablation must be fully transmural to ensure that the atrial wall can no longer conduct electrical impulses to the adjacent atrial tissue. Indeed, the success of catheter ablation of AF is dependent on ensuring transmurality of the RF lesions [9], which in turn depends on several parameters such as contact force between the catheter and the endocardium, the power, the duration of RF, and also the amount of epicardial fat [10]. Suárez et al. investigated the physics of the histological lesion induced by RF current in an *in vitro* model of atrial tissue according to the thickness of EF [11]. They observed that at constant voltage, increasing fat thickness implied a decrease in the temperature in the atrial tissue, and also a decrease in the depth of the lesion in the atrial wall. They observed similar results when a constant temperature was delivered by the active electrode. Furthermore, the increase in epicardial fat thickness is directly related to the increase in the maximal temperature distribution. Our findings are in line with these reports, notably the fact that the ablation procedure is longer and more complex when epicardial fat is more abundant. The fact that the association between epicardial fat and RF duration was not significant in multivariate analysis could be first explained by a relative long standardized consolidation application after elimination of the positive component of the atrial electrogram for each lesion; second, by the fact that a larger volume of the LA (by consequence a larger surface to treat) needs more radiofrequency energy delivery. The collinearity between the total volume of EF and the LA volume was not high (p < 0.0001; R^2^ = 0.176). This compels us to take the volume of the LA into account into the multivariate model.

It is now well established that obesity and visceral fat play a major role in the onset of AF.

Visceral fat (which includes EF) has more pronounced metabolic properties than subcutaneous fat. Epicardial fat shares a common origin with visceral fat, namely they both originate from the splanchnopleuric mesoderm, whereas paracardial fat is derived from the primitive thoracic mesenchyme [4]. Thanassoulis et al. showed that pericardial fat was associated with prevalent AF, but not paracardial, intrathoracic or visceral abdominal fat [12]. EF contributes to AF via several mechanisms. Apart from its beneficial effects on thermoregulation and energy reserves, it plays a role in the onset of arrhythmia through a phenomenon of contiguity; Indeed, EF comprises free fatty acids that can infiltrate adjacent subepicardial and myocardial tissue, inducing fibrosis [13]. Fibrosis in turn contributes to structural remodelling, creating a substrate for AF, and also to functional remodelling, leading to heterogeneous conduction and the formation of micro-reentry circuits. Fibrosis is also mediated by an inflammatory immune response involving CD4+ and CD8+ lymphocytes [14].

EF also acts via the endocrine and paracrine pathways through the production of pro-fibrotic cytokines such as activin A, a member of the TGF-beta family [15], as well as adipocytokines, which can infiltrate the subepicardial tissue. In our study, periventricular epicardial fat seemed to be as related to the duration of RF delivery as the periatrial epicardial fat is. This finding highlights the involvement of the paracrine and endocrine pathways through which epicardial fat contributes to the genesis of AF, as compared to other mechanisms relying on contiguity. The fact that the association between epicardial fat volume and recurrence at 1 year of symptomatic FA was not significant could be explained by a lack of power, a different definition of AF recurrence or a different holter monitoring frequency according to other studies.

There are numerous methods to quantify epicardial fat: among available approaches, magnetic resonance imaging (MRI) offers the best spatial resolution and as such, is considered as the gold standard imaging exam to evaluate and quantify the adipose tissue. Indeed, it is the only imaging modality that is validated for volumetric quantification of EF [16]. MRI presents the advantage of being non-irradiating but remains less accessible and more expensive than other imaging techniques. Transthoracic echocardiography is more easily accessible and less costly, but due to its low spatial resolution, the measure of EF alone has poor reproducibility in routine practice. Consequently, only the measure of EF thickness is possible and validated in 2D [17]. In the literature, the most commonly used imaging technique to quantify epicardial fat is CT scanning. Our study also used CT scans to evaluate fat volume, because our patients underwent systematic CT scan prior to the ablation procedure. Measurement methods from CT scans can be manual, as in our study, or semi-automatic. The major drawback of the CT scan is the exposure to ionizing radiation [18]. However, its excellent spatial resolution ensures reproducible measurement of the thickness, area or volume of fat, and also enables estimation of the distribution of epicardial fat [19]. The majority of studies of EF have only focused on periventricular fat [4]. Gaborit et al. reported that periatrial fat is atypical adipose tissue that has different characteristics to those of periventricular or pericoronary fat, expressing genes involved in oxidative phosphorylation and calcium signalling, making it thus more pathogenic [20]. Other studies reported no difference in the strength of the association between periatrial and periventricular fat in the incidence of AF [4]. These disparate findings prompted us to measure EF at both levels in the present study.

The estimation of EF volume on CT images prior to AF ablation is nowadays not systematic in current practice. Systematic quantification and distribution of epicardial fat implies a longer post processing work for radiologists but brings needful information to the electrophysiologist to target potential sites of interest. It also could help identifying patients who will yield greater benefit from catheter ablation of AF and stay free longer from recurrences [8].

### 4.1 Study limitations

This study has some limitations. Firstly, CT scan quantification of EF were all performed by a single radiologist. Although there is small operator dependency on CT scan, it would have been preferable to have two readings by independent observers to limit any potential discrepancies between volume measures. Second, this was a retrospective study, and is thus subject to the inherent bias of this type of investigation, such as missing data or a low level of evidence.

## 5 Conclusion

Our study showed a significant association between total, periatrial, and periventricular epicardial fat volume, and the duration of RF energy application during catheter ablation of AF. This relation was no longer significant by multivariate analysis meaning that epicardial fat may be a marker, but not an independent factor, of ablation complexity.

## Declaration of Competing Interest

The authors declared that there is no conflict of interest.
